# Retraction: Involvement of FOXO Transcription Factors, TRAIL-FasL/Fas, and Sirtuin Proteins Family in Canine Coronavirus Type II-Induced Apoptosis

**DOI:** 10.1371/journal.pone.0324091

**Published:** 2025-05-06

**Authors:** The PLOS One Editors

After this article [1] was published, concerns were raised regarding results presented in Figures 1, 2, 3, 7, 8, 9, 10, and 11.

Specifically:

- There appears to be vertical and horizontal discontinuities in the background surrounding lanes of the following panels:
  - Figure 2A SIRT3
  - Figure 7A β-actin
  - Figure 8A β-actin
- There appears to be similarities between lanes within the following panels:
  - Figure 1A: SIRT1, FOXO3A, FOXO1, and β-actin
  - Figure 2A: SIRT4
  - Figure 3A: bax, bcl-2, and β-actin
  - Figure 7A: TIM50 (mitochondria)
  - Figure 9A: bax (mitochondria), bax (cytosol), and bcl-2 (cytosol)
  - Figure 10A: SIRT1, SIRT3, and β-actin
  - Figure 11A: FOXO3A and FOXO1
- The following results appear more similar than would be expected from independent results:
  - Figure 1A SIRT1 lanes 1, 2, and 7 of [1] and Figure 4B bax lanes 1, 2, and 7 of [2] respectively
  - Figure 1A FOXO3A lanes 2-6 of [1] and Figure 4B p27 lanes 2-6 of [2] respectively
  - Figure 1A FOXO1 lanes 1-4 of [1] and Figure 4B CDK-2 lanes 2-5 of [2] respectively
  - Figure 1A FOXO1 lane 6 of [1], Figure 1A FOXO1 lane 7 of [1], and Figure 4B CDK-2 lane 7 of [2]
  - Figure 1A β-actin lanes 1-3 and 6-7 of [1] and Figure 4B β-actin lanes 1-3 and 5-6 of [2] respectively
  - Figure 1A β-actin lane 4 of [1], Figure 1A β-actin lane 5 of [1], and Figure 4B β-actin lane 4 of [2]

The authors’ responses did not resolve the concerns with this article.

In light of the above concerns that question the integrity and reliability of the reported results and conclusions, the *PLOS One* Editors retract this article.

All authors did not respond to the final editorial decision.

The Figure 1A results report material that appears similar to results previously reported in [2] published in 2006 by The American Society of Hematology, which is not offered under a CC BY license. Due to restrictions that apply to the original article’s license, Figure 1A is excluded from the PLOS article’s [1] CC BY 4.0 license.
